# 3,6-Dimethyl-*N* ^1^,*N* ^4^-bis­(pyridin-2-yl)-1,2,4,5-tetra­zine-1,4-dicarboxamide

**DOI:** 10.1107/S1600536812005405

**Published:** 2012-02-17

**Authors:** Guo-Wu Rao, Yan-Mei Guo, Qun Shen

**Affiliations:** a College of Pharmaceutical Science, Zhejiang University of Technology, Hangzhou 310014, People’s Republic of China; b Hangzhou Institute of Calibration and Testing for Quality and Technical Supervision, Hangzhou 310019, People’s Republic of China

## Abstract

In the title mol­ecule, C_16_H_16_N_8_O_2_, four atoms of the tetra­zine ring are coplanar, with the largest deviation from the plane being 0.0236 (12) Å; the other two atoms of the tetra­zine ring deviate on the same side from this plane by 0.320 (4) and 0.335 (4) Å. Therefore, the central tetra­zine ring exhibits a boat conformation. The dihedral angles between the mean plane of the four coplanar atoms of the tetrazine ring and the two pyridine rings are 26.22 (10) and 6.97 (5)°. The two pyridine rings form a dihedral angle of 31.27 (8)°. In the molecule, there are a number of short C—H⋯O interactions. In the crystal, molecules are linked *via* a C—H⋯O interaction to form zigzag chains propagating along the [010] direction.

## Related literature

For the activities of 1,2,4,5-tetra­zine derivatives in chemical reactions, see: Domingo *et al.* (2009 ▶); Lorincz *et al.* (2010 ▶). For biological activities in 1,2,4,5-tetra­zine derivatives, see: Eremeev *et al.* (1978 ▶, 1980 ▶); Neunhoeffer (1984 ▶); Sauer (1996 ▶). For anti­tumor activities of 1,2,4,5-tetra­zine derivatives, see: Hu *et al.* (2002 ▶, 2004 ▶); Rao & Hu (2005 ▶, 2006 ▶). For typical bond lengths for C=N double and C—N and N—N single bonds, see: Allen *et al.* (1987 ▶). For the synthesis of the title compound, see: Hu *et al.* (2004 ▶); Skorianetz & Kováts (1970 ▶, 1971 ▶); Sun *et al.* (2003 ▶).
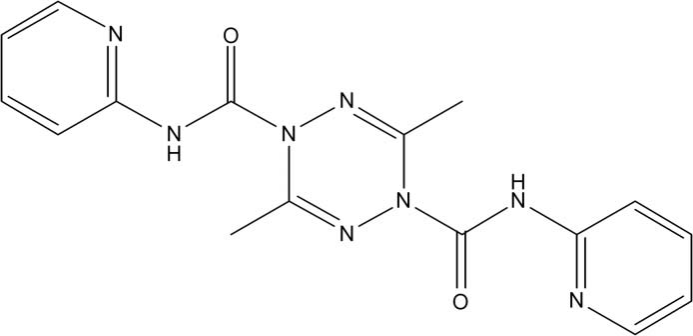


## Experimental

### 

#### Crystal data

C_16_H_16_N_8_O_2_
*M* *_r_* = 352.37
Monoclinic, 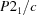

*a* = 11.753 (2) Å
*b* = 20.081 (4) Å
*c* = 7.2012 (14) Å
β = 96.273 (3)°
*V* = 1689.4 (6) Å^3^
*Z* = 4
Mo *K*α radiation
μ = 0.10 mm^−1^
*T* = 298 K
0.36 × 0.23 × 0.20 mm

#### Data collection

Bruker SMART CCD area-detector diffractometer
Absorption correction: multi-scan (*SADABS*; Bruker, 1997 ▶) *T*_min_ = 0.965, *T*_max_ = 0.981
7032 measured reflections
2985 independent reflections
2430 reflections with *I* > 2σ(*I*)
*R*_int_ = 0.024

#### Refinement

*R*[*F* ^2^ > 2σ(*F* ^2^)] = 0.061
*wR*(*F* ^2^) = 0.182
*S* = 1.07
2985 reflections
236 parameters
H-atom parameters constrained
Δρ_max_ = 0.44 e Å^−3^
Δρ_min_ = −0.33 e Å^−3^

### 

Data collection: *SMART* (Bruker, 1997 ▶); cell refinement: *SAINT* (Bruker, 1997 ▶); data reduction: *SAINT*; program(s) used to solve structure: *SHELXS97* (Sheldrick, 2008 ▶); program(s) used to refine structure: *SHELXL97* (Sheldrick, 2008 ▶); molecular graphics: *ORTEP-3 for Windows* (Farrugia, 1997 ▶); software used to prepare material for publication: *WinGX* (Farrugia, 1999 ▶).

## Supplementary Material

Crystal structure: contains datablock(s) I, global. DOI: 10.1107/S1600536812005405/zj2056sup1.cif

Structure factors: contains datablock(s) I. DOI: 10.1107/S1600536812005405/zj2056Isup2.hkl

Supplementary material file. DOI: 10.1107/S1600536812005405/zj2056Isup3.cdx

Supplementary material file. DOI: 10.1107/S1600536812005405/zj2056Isup4.cml

Additional supplementary materials:  crystallographic information; 3D view; checkCIF report

## Figures and Tables

**Table 1:** Hydrogen-bond geometry (Å, °)

*D*—H⋯*A*	*D*—H	H⋯*A*	*D*⋯*A*	*D*—H⋯*A*
C1—H1*A*⋯O2	0.96	2.07	2.778 (4)	130
C2—H2*A*⋯O1	0.96	2.09	2.777 (4)	127
C8—H8⋯O1	0.93	2.31	2.886 (3)	120
C16—H16⋯O1^i^	0.93	2.39	3.229 (3)	150

Symmetry code: (i) 
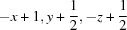
.

**Fig. 1.**
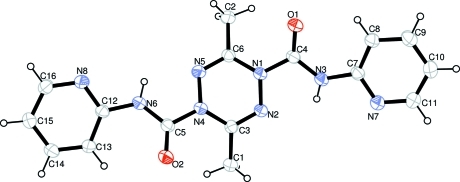
The structure of (I), shown with 30% probability displacement ellipsoids.

**Fig. 2.**
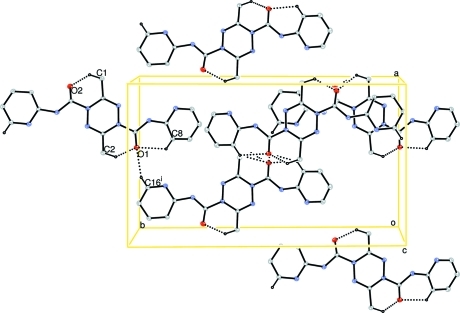
A portion of the crystal packing of (I). Hydrogen bonds are shown as dashed lines. H atoms not involved in hydrogen bonding were omitted for clarity.

## supplementary crystallographic information

### Comment

Tetrazine derivatives have high activities of chemical reaction (Domingo *et al.*, 2009; Lorincz *et al.*, 2010), and have been widely used in pesticides and medicines (Eremeev *et al.*, 1978, 1980; Neunhoeffer, 1984; Sauer, 1996). In a continuation of our studies of antitumor activities in 1,2,4,5-tetrazine derivatives (Hu *et al.*, 2002, 2004; Rao & Hu, 2005, 2006), we have obtained a colourless crystalline compound, (I). However, IR, NMR, and MS studies failed to prove whether the substituted groups of the nitrogen are located at the 1,4 or 1,2 position. The structure was confirmed by single-crystal X-ray diffraction.

The molecular structure of (I) is illustrated in Fig. 1. The N2═C3 [1.279 (3) Å] and N5═C6 [1.278 (3) Å] bonds correspond to typical double bonds of C═N, and the C3—N4 [1.395 (3) Å], N4—N5 [1.419 (3) Å], C6—N1 [1.388 (3) Å] and N1—N2 [1.420 (3) Å] bond lengths correspond to typical single bonds (Allen *et al.*, 1987). Therefore, the tetrazine ring is the 1,4-dihydro structure with the N-substituted groups at the 1,4-positions and not the 1,2-positions, the compound being 3,6-dimethyl-*N*^1^,*N*^4^-di(pyridin-2-yl)-1,2,4,5-tetrazine-1,4-dicarboxamide, rather than the 3,6-dimethyl-*N*^1^,*N*^2^-di(pyridin-2-yl)-1,2,4,5-tetrazine-1,2-dicarboxamide.

In (I), atoms N2, C3, N5 and C6 are coplanar, with the largest deviation from this plane being ±0.0236 (12) Å. Atoms N1 and N4 deviate from this plane by -0.3202 (35) and -0.3345 (36) Å, respectively. The dihedral angle between the N2/C3/N5/C6 plane and the N1/N2/C6 plane is 26.25 (18)°, and between the N2/C3/N5/C6 plane and the N4/N5/C3 plane is 27.18 (19)°. Therefore, the central six-member ring of the compound, the tetrazine ring, has an obvious boat conformation. The dihedral angles between the N2/C3/N5/C6 plane and the two pyridyl rings are 26.22 (10) and 6.97 (5)°, respectively. And two pyridyl rings form a dihedral angle of 31.27 (8)°. In the molecule, there are a number of short C—H···O interactions. In the crystal, molecules are linked *via* a C—H···O interaction to form zigzag chains propagating along the [010] direction.

### Experimental

The title compound was prepared according to the procedure of Hu *et al.* (2004), Sun *et al.* (2003) and Skorianetz & Kováts (1970, 1971). A saturated solution of the compound in ethanol and dichloromethane (4:1, *v*/*v*) of 298 K was concentrated gradually at room temperature to afford colourless blocks.

### Refinement

H atoms were included in calculated positions and refined using a riding model. H atoms were given isotropic displacement parameters equal to 1.2 (or 1.5 for methyl H atoms) times the equivalent isotropic displacement parameters of their parent atoms, and C—H distances were set to 0.96 Å for methyl H atoms and 0.93 Å for pyridyl H atoms, while N—H distances were set to 0.86 Å.

### Crystal data

**Table:**

C_16_H_16_N_8_O_2_	*Z* = 4
*M**_r_* = 352.37	*F*(000) = 736
Monoclinic, *P*2_1_/*c*	*D*_x_ = 1.385 Mg m^−^^3^
Hall symbol: -P 2ybc	Mo *K*α radiation, λ = 0.71073 Å
*a* = 11.753 (2) Å	θ = 3.0–28.2°
*b* = 20.081 (4) Å	µ = 0.10 mm^−^^1^
*c* = 7.2012 (14) Å	*T* = 298 K
β = 96.273 (3)°	Block, colourless
*V* = 1689.4 (6) Å^3^	0.36 × 0.23 × 0.20 mm

### Data collection

**Table:**

Bruker SMART CCD area-detector diffractometer	2985 independent reflections
Radiation source: fine-focus sealed tube	2430 reflections with *I* > 2σ(*I*)
Graphite monochromator	*R*_int_ = 0.024
φ and ω scans	θ_max_ = 25.0°, θ_min_ = 1.7°
Absorption correction: multi-scan (*SADABS*; Bruker, 1997)	*h* = −13→11
*T*_min_ = 0.965, *T*_max_ = 0.981	*k* = −23→22
7032 measured reflections	*l* = −8→8

### Refinement

**Table:**

Refinement on *F*^2^	Secondary atom site location: difference Fourier map
Least-squares matrix: full	Hydrogen site location: inferred from neighbouring sites
*R*[*F*^2^ > 2σ(*F*^2^)] = 0.061	H-atom parameters constrained
*wR*(*F*^2^) = 0.182	*w* = 1/[σ^2^(*F*_o_^2^) + (0.0986*P*)^2^ + 0.7423*P*] where *P* = (*F*_o_^2^ + 2*F*_c_^2^)/3
*S* = 1.07	(Δ/σ)_max_ < 0.001
2985 reflections	Δρ_max_ = 0.44 e Å^−^^3^
236 parameters	Δρ_min_ = −0.33 e Å^−^^3^
0 restraints	Extinction correction: *SHELXL97* (Sheldrick, 2008), Fc^*^=kFc[1+0.001xFc^2^λ^3^/sin(2θ)]^-1/4^
Primary atom site location: structure-invariant direct methods	Extinction coefficient: 0.012 (2)

### Special details

**Table:**

Geometry. All e.s.d.'s (except the e.s.d. in the dihedral angle between two l.s. planes) are estimated using the full covariance matrix. The cell e.s.d.'s are taken into account individually in the estimation of e.s.d.'s in distances, angles and torsion angles; correlations between e.s.d.'s in cell parameters are only used when they are defined by crystal symmetry. An approximate (isotropic) treatment of cell e.s.d.'s is used for estimating e.s.d.'s involving l.s. planes.
Refinement. Refinement of *F*^2^ against ALL reflections. The weighted *R*-factor *wR* and goodness of fit *S* are based on *F*^2^, conventional *R*-factors *R* are based on *F*, with *F* set to zero for negative *F*^2^. The threshold expression of *F*^2^ > σ(*F*^2^) is used only for calculating *R*-factors(gt) *etc*. and is not relevant to the choice of reflections for refinement. *R*-factors based on *F*^2^ are statistically about twice as large as those based on *F*, and *R*-factors based on ALL data will be even larger.

### Fractional atomic coordinates and isotropic or equivalent isotropic displacement parameters (Å^2^)

**Table:**

	*x*	*y*	*z*	*U*_iso_*/*U*_eq_	
N1	0.70522 (17)	0.06533 (10)	0.3165 (3)	0.0460 (5)	
O2	0.94498 (16)	0.25045 (9)	0.2447 (3)	0.0587 (6)	
O1	0.54879 (15)	0.00173 (9)	0.2281 (3)	0.0618 (6)	
N4	0.80771 (16)	0.17897 (10)	0.3261 (3)	0.0464 (5)	
N5	0.69810 (17)	0.17708 (10)	0.3912 (3)	0.0481 (6)	
N2	0.82657 (17)	0.06420 (10)	0.3487 (3)	0.0480 (6)	
N6	0.78462 (18)	0.29223 (10)	0.3540 (3)	0.0489 (6)	
H6	0.7217	0.2801	0.3948	0.059*	
N8	0.70889 (18)	0.39638 (11)	0.3514 (3)	0.0529 (6)	
C12	0.8038 (2)	0.36064 (12)	0.3445 (3)	0.0434 (6)	
C3	0.8743 (2)	0.12142 (12)	0.3484 (4)	0.0448 (6)	
N7	0.7973 (2)	−0.14331 (12)	0.1576 (4)	0.0649 (7)	
N3	0.72486 (18)	−0.04631 (10)	0.2546 (3)	0.0532 (6)	
H3	0.7933	−0.0403	0.3073	0.064*	
C6	0.6486 (2)	0.12036 (12)	0.3784 (3)	0.0432 (6)	
C4	0.6513 (2)	0.00504 (12)	0.2642 (4)	0.0449 (6)	
C5	0.8541 (2)	0.24270 (12)	0.3062 (4)	0.0453 (6)	
C7	0.7024 (2)	−0.10818 (12)	0.1687 (4)	0.0470 (6)	
C2	0.5324 (2)	0.11538 (14)	0.4433 (4)	0.0570 (7)	
H2A	0.5046	0.0706	0.4263	0.086*	
H2B	0.4811	0.1453	0.3721	0.086*	
H2C	0.5369	0.1270	0.5733	0.086*	
C13	0.9103 (2)	0.38885 (13)	0.3336 (4)	0.0503 (7)	
H13	0.9753	0.3625	0.3325	0.060*	
C8	0.5943 (2)	−0.13104 (13)	0.1018 (4)	0.0538 (7)	
H8	0.5294	−0.1055	0.1130	0.065*	
C16	0.7187 (2)	0.46207 (14)	0.3430 (4)	0.0585 (8)	
H16	0.6530	0.4876	0.3476	0.070*	
C9	0.5865 (3)	−0.19228 (15)	0.0190 (4)	0.0637 (8)	
H9	0.5153	−0.2090	−0.0276	0.076*	
C14	0.9165 (2)	0.45670 (14)	0.3243 (5)	0.0590 (8)	
H14	0.9866	0.4773	0.3156	0.071*	
C1	1.0012 (2)	0.12417 (14)	0.3833 (5)	0.0599 (8)	
H1A	1.0262	0.1696	0.3778	0.090*	
H1B	1.0340	0.0985	0.2900	0.090*	
H1C	1.0255	0.1062	0.5047	0.090*	
C15	0.8196 (2)	0.49461 (14)	0.3279 (4)	0.0617 (8)	
H15	0.8225	0.5408	0.3204	0.074*	
C11	0.7852 (3)	−0.20271 (15)	0.0760 (6)	0.0745 (10)	
H11	0.8509	−0.2278	0.0673	0.089*	
C10	0.6832 (3)	−0.22915 (15)	0.0042 (5)	0.0677 (9)	
H10	0.6792	−0.2708	−0.0528	0.081*	

### Atomic displacement parameters (Å^2^)

**Table:**

	*U*^11^	*U*^22^	*U*^33^	*U*^12^	*U*^13^	*U*^23^
N1	0.0367 (11)	0.0442 (12)	0.0563 (13)	−0.0043 (8)	0.0015 (9)	−0.0017 (9)
O2	0.0514 (11)	0.0516 (11)	0.0763 (14)	−0.0098 (8)	0.0211 (10)	−0.0047 (9)
O1	0.0400 (11)	0.0556 (12)	0.0884 (15)	−0.0066 (8)	0.0015 (10)	−0.0091 (10)
N4	0.0383 (11)	0.0449 (12)	0.0569 (13)	−0.0051 (9)	0.0096 (9)	−0.0016 (9)
N5	0.0416 (12)	0.0473 (12)	0.0564 (13)	−0.0014 (9)	0.0106 (10)	0.0033 (10)
N2	0.0385 (11)	0.0458 (12)	0.0589 (14)	−0.0031 (9)	0.0014 (9)	−0.0054 (10)
N6	0.0411 (11)	0.0452 (12)	0.0612 (14)	−0.0053 (9)	0.0091 (10)	0.0018 (10)
N8	0.0386 (12)	0.0528 (13)	0.0662 (15)	−0.0012 (9)	0.0005 (10)	−0.0054 (11)
C12	0.0392 (13)	0.0466 (14)	0.0435 (14)	−0.0031 (10)	−0.0001 (10)	−0.0015 (10)
C3	0.0423 (13)	0.0445 (14)	0.0476 (14)	−0.0025 (10)	0.0042 (11)	−0.0062 (11)
N7	0.0469 (14)	0.0496 (13)	0.097 (2)	−0.0010 (10)	0.0032 (13)	−0.0065 (13)
N3	0.0397 (12)	0.0461 (12)	0.0713 (16)	−0.0053 (9)	−0.0047 (10)	−0.0034 (11)
C6	0.0417 (13)	0.0430 (13)	0.0446 (14)	−0.0024 (10)	0.0037 (10)	0.0049 (10)
C4	0.0391 (14)	0.0456 (14)	0.0498 (15)	−0.0056 (10)	0.0033 (11)	0.0033 (11)
C5	0.0424 (14)	0.0458 (14)	0.0475 (14)	−0.0053 (11)	0.0048 (11)	−0.0032 (11)
C7	0.0438 (14)	0.0422 (14)	0.0546 (16)	−0.0047 (11)	0.0029 (11)	0.0049 (11)
C2	0.0475 (15)	0.0543 (16)	0.0718 (19)	−0.0021 (12)	0.0180 (13)	0.0038 (13)
C13	0.0383 (13)	0.0482 (15)	0.0634 (17)	−0.0017 (11)	0.0012 (12)	−0.0047 (12)
C8	0.0452 (15)	0.0504 (15)	0.0643 (18)	−0.0017 (11)	−0.0011 (13)	−0.0011 (13)
C16	0.0480 (15)	0.0530 (16)	0.074 (2)	0.0060 (12)	0.0023 (13)	−0.0084 (14)
C9	0.0574 (17)	0.0631 (18)	0.068 (2)	−0.0102 (14)	−0.0051 (14)	−0.0099 (15)
C14	0.0464 (15)	0.0530 (16)	0.079 (2)	−0.0108 (12)	0.0120 (14)	−0.0075 (14)
C1	0.0422 (15)	0.0531 (16)	0.083 (2)	−0.0020 (12)	0.0027 (14)	−0.0067 (14)
C15	0.0600 (18)	0.0449 (15)	0.081 (2)	−0.0065 (13)	0.0130 (15)	−0.0078 (14)
C11	0.0591 (19)	0.0521 (17)	0.113 (3)	0.0049 (14)	0.0123 (18)	−0.0106 (17)
C10	0.070 (2)	0.0518 (17)	0.081 (2)	−0.0028 (15)	0.0032 (16)	−0.0148 (15)

### Geometric parameters (Å, º)

**Table:**

N1—C6	1.388 (3)	C6—C2	1.494 (3)
N1—C4	1.399 (3)	C7—C8	1.387 (4)
N1—N2	1.420 (3)	C2—H2A	0.9600
O2—C5	1.210 (3)	C2—H2B	0.9600
O1—C4	1.206 (3)	C2—H2C	0.9600
N4—C3	1.395 (3)	C13—C14	1.367 (4)
N4—C5	1.405 (3)	C13—H13	0.9300
N4—N5	1.419 (3)	C8—C9	1.366 (4)
N5—C6	1.278 (3)	C8—H8	0.9300
N2—C3	1.279 (3)	C16—C15	1.369 (4)
N6—C5	1.356 (3)	C16—H16	0.9300
N6—C12	1.395 (3)	C9—C10	1.370 (4)
N6—H6	0.8600	C9—H9	0.9300
N8—C16	1.326 (4)	C14—C15	1.372 (4)
N8—C12	1.332 (3)	C14—H14	0.9300
C12—C13	1.384 (3)	C1—H1A	0.9600
C3—C1	1.487 (3)	C1—H1B	0.9600
N7—C7	1.329 (3)	C1—H1C	0.9600
N7—C11	1.331 (4)	C15—H15	0.9300
N3—C4	1.353 (3)	C11—C10	1.360 (4)
N3—C7	1.400 (3)	C11—H11	0.9300
N3—H3	0.8600	C10—H10	0.9300
			
C6—N1—C4	123.8 (2)	C6—C2—H2B	109.5
C6—N1—N2	117.91 (19)	H2A—C2—H2B	109.5
C4—N1—N2	116.61 (19)	C6—C2—H2C	109.5
C3—N4—C5	123.2 (2)	H2A—C2—H2C	109.5
C3—N4—N5	117.25 (19)	H2B—C2—H2C	109.5
C5—N4—N5	115.78 (19)	C14—C13—C12	117.7 (2)
C6—N5—N4	115.0 (2)	C14—C13—H13	121.2
C3—N2—N1	114.7 (2)	C12—C13—H13	121.2
C5—N6—C12	127.2 (2)	C9—C8—C7	117.7 (3)
C5—N6—H6	116.4	C9—C8—H8	121.1
C12—N6—H6	116.4	C7—C8—H8	121.1
C16—N8—C12	117.2 (2)	N8—C16—C15	124.0 (3)
N8—C12—C13	123.2 (2)	N8—C16—H16	118.0
N8—C12—N6	112.9 (2)	C15—C16—H16	118.0
C13—C12—N6	123.9 (2)	C8—C9—C10	120.2 (3)
N2—C3—N4	120.3 (2)	C8—C9—H9	119.9
N2—C3—C1	117.7 (2)	C10—C9—H9	119.9
N4—C3—C1	121.9 (2)	C13—C14—C15	120.2 (3)
C7—N7—C11	116.9 (2)	C13—C14—H14	119.9
C4—N3—C7	127.5 (2)	C15—C14—H14	119.9
C4—N3—H3	116.2	C3—C1—H1A	109.5
C7—N3—H3	116.2	C3—C1—H1B	109.5
N5—C6—N1	120.2 (2)	H1A—C1—H1B	109.5
N5—C6—C2	117.5 (2)	C3—C1—H1C	109.5
N1—C6—C2	122.3 (2)	H1A—C1—H1C	109.5
O1—C4—N3	125.2 (2)	H1B—C1—H1C	109.5
O1—C4—N1	121.2 (2)	C16—C15—C14	117.7 (3)
N3—C4—N1	113.6 (2)	C16—C15—H15	121.2
O2—C5—N6	125.4 (2)	C14—C15—H15	121.2
O2—C5—N4	121.5 (2)	N7—C11—C10	124.4 (3)
N6—C5—N4	113.0 (2)	N7—C11—H11	117.8
N7—C7—C8	123.1 (3)	C10—C11—H11	117.8
N7—C7—N3	112.3 (2)	C11—C10—C9	117.6 (3)
C8—C7—N3	124.6 (2)	C11—C10—H10	121.2
C6—C2—H2A	109.5	C9—C10—H10	121.2
			
C3—N4—N5—C6	−32.6 (3)	C6—N1—C4—N3	−164.0 (2)
C5—N4—N5—C6	168.6 (2)	N2—N1—C4—N3	0.8 (3)
C6—N1—N2—C3	−31.4 (3)	C12—N6—C5—O2	−0.8 (4)
C4—N1—N2—C3	162.8 (2)	C12—N6—C5—N4	−177.8 (2)
C16—N8—C12—C13	1.6 (4)	C3—N4—C5—O2	25.5 (4)
C16—N8—C12—N6	−179.9 (2)	N5—N4—C5—O2	−177.0 (2)
C5—N6—C12—N8	162.7 (2)	C3—N4—C5—N6	−157.3 (2)
C5—N6—C12—C13	−18.8 (4)	N5—N4—C5—N6	0.1 (3)
N1—N2—C3—N4	2.8 (3)	C11—N7—C7—C8	0.7 (5)
N1—N2—C3—C1	179.3 (2)	C11—N7—C7—N3	−179.2 (3)
C5—N4—C3—N2	−173.6 (2)	C4—N3—C7—N7	169.7 (3)
N5—N4—C3—N2	29.3 (3)	C4—N3—C7—C8	−10.2 (4)
C5—N4—C3—C1	10.0 (4)	N8—C12—C13—C14	−1.9 (4)
N5—N4—C3—C1	−147.1 (3)	N6—C12—C13—C14	179.8 (3)
N4—N5—C6—N1	4.2 (3)	N7—C7—C8—C9	−0.8 (4)
N4—N5—C6—C2	−179.5 (2)	N3—C7—C8—C9	179.1 (3)
C4—N1—C6—N5	−167.2 (2)	C12—N8—C16—C15	−0.1 (4)
N2—N1—C6—N5	28.1 (3)	C7—C8—C9—C10	0.1 (5)
C4—N1—C6—C2	16.6 (4)	C12—C13—C14—C15	0.7 (4)
N2—N1—C6—C2	−148.0 (2)	N8—C16—C15—C14	−1.0 (5)
C7—N3—C4—O1	13.6 (5)	C13—C14—C15—C16	0.7 (5)
C7—N3—C4—N1	−164.6 (2)	C7—N7—C11—C10	0.1 (5)
C6—N1—C4—O1	17.6 (4)	N7—C11—C10—C9	−0.7 (6)
N2—N1—C4—O1	−177.6 (2)	C8—C9—C10—C11	0.6 (5)

### Hydrogen-bond geometry (Å, º)

**Table:**

*D*—H···*A*	*D*—H	H···*A*	*D*···*A*	*D*—H···*A*
C1—H1*A*···O2	0.96	2.07	2.778 (4)	130
C2—H2*A*···O1	0.96	2.09	2.777 (4)	127
C8—H8···O1	0.93	2.31	2.886 (3)	120
C16—H16···O1^i^	0.93	2.39	3.229 (3)	150

Symmetry code: (i) −*x*+1, *y*+1/2, −*z*+1/2.
